# Relation of Nonrecurrent Laryngeal Nerve with Zuckerkandl's Tubercle

**DOI:** 10.1155/2020/2459321

**Published:** 2020-08-04

**Authors:** Carlos Alberto Ferreira de Freitas, Maria Margarida Morena Domingos Levenhagen, Isabela Salvador Constantino, Amauri Ferrari Paroni, Marcelo Resende Martins

**Affiliations:** ^1^Department of Head Neck Surgery, University Hospital, Federal University of Mato Grosso do Sul Medical School, Brazil; ^2^Federal University of Mato Grosso do Sul Medical School, Brazil

## Abstract

The nonrecurrent laryngeal nerve (NRLN) is a rare anatomical variation of the recurrent laryngeal nerve (RLN) that may hinder the identification and preservation of this nerve during surgery and is associated with increased iatrogenic risks. Zuckerkandl's tubercle (ZT) is considered a useful reference for locating the RLN during thyroid surgery. We report the case of an asymptomatic patient with a 23 mm uninodular goitre suspicious for cancer. Ultrasound examination showed a hypoechoic nodule with regular contours and microcalcifications. The patient had normal thyroid-stimulating hormone and thyroxine levels, and aspiration biopsy was suspicious for follicular cancer. She was treated with total thyroidectomy after the intraoperative examination confirmed the presence of a papillary thyroid carcinoma. The standard approach to the RLN below the inferior thyroid artery was used on both sides. The nerve displayed anatomical variation in the nonrecurrent form (NRLN) on the right side and was associated with another variation that was not found in the consulted literature. It was completely surrounded by thyroid tissue in the region of ZT, and the surgeon was forced to remove it from within the thyroid tissue. This combination of anatomical variations seems to be quite rare. Knowledge of the anatomy of the RLN and its variations, as well as its identification and careful dissection, is essential to avoid injury to the nerve during surgical procedures.

## 1. Introduction

The nonrecurrent laryngeal nerve (NRLN) is an anatomical variation of the recurrent laryngeal nerve (RLN), which innervates all intrinsic muscles of the larynx except for the cricothyroid muscle [1]. The NRLN occurs in 0.7% of individuals, usually on the right side, and may be associated with the variation of a right subclavian artery with left-side origin that travels behind the oesophagus (arteria lusoria) [2]. Although rare, NRLN is often associated with increased risk of iatrogenic surgical injury due to difficulties with its identification and preservation [2].

Zuckerkandl's tubercle (ZT) is a lateral projection of the thyroid gland that is present in most people (68%), and when present, it is a useful landmark for the identification of the RLN during thyroid and parathyroid surgery [3]. The RLN is located posterior to the ZT, near the site at which the RLN enters the larynx [3].

To minimize the risk of injury and improve the success rate of procedures, head and neck surgeons should be aware of the embryological development and all anatomical variations of the thyroid gland, parathyroid gland, and adjacent anatomical structures [4].

In this article, we report the case of a patient with an anatomical variation of the NRLN not found in other publications. Zuckerkandl's tubercle completely enveloped the nerve, forcing the surgeon to remove it from within the thyroid tissue. This case report is part of a morphological study submitted to and approved by the Research Ethics Committee of the Federal University of Mato Grosso do Sul (protocol no. 29/05).

## 2. Case Report

**Figure 1:**
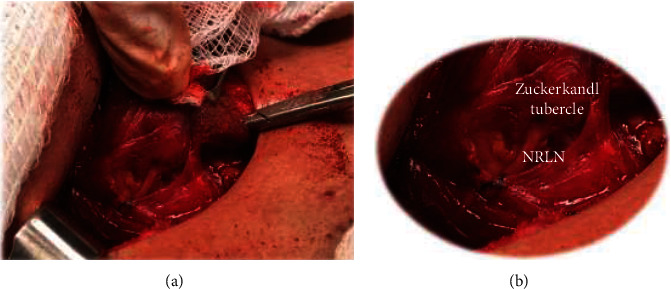
(a) Intraoperative picture evidencing a nonrecurrent laryngeal nerve with a path through the Zuckerkandl tubercle (ZT). (b) Enlarged intraoperative picture, evidencing internal nerve path in the ZT.

Female patient, aged 55 years, with a history of nodular goitre for two months, diagnosed on routine ultrasound (US) examination. On physical examination, there were no palpable nodules, although cervical US revealed a single hypoechoic nodule (19 mm in diameter) in the left lobe. Doppler showed the presence of peripheral and central flows. US-guided thin-needle biopsy was suspicious for follicular cancer. The patient had a history of breast cancer that had been diagnosed and treated nine years prior and was being followed up by an oncologist; she was also undergoing clinical treatment for hypertension, lactose intolerance, and psoriasis. The patient was euthyroid. A new US was performed during the preoperative period and showed a single hypoechoic nodule (now 23 mm in diameter two months after the first US) in the left lobe, with a regular contour, microcalcifications, and the absence of lymphadenomegaly. The left lobe was accessed using the conventional technique to determine whether the RLN was below the inferior thyroid artery (ITA) and followed until its entrance into the larynx. The left lobe was removed without complications, preserving the laryngeal nerves and the parathyroid glands. Frozen section biopsy revealed papillary carcinoma, and thyroid resection was indicated for this patient. To access the right lobe, the standard approach to the nerve below the ITA was also used, but the right RLN was not found along its usual course, and the dissection had to be advanced in the cephalic direction. An NRLN was identified and exposed parallel to the ITA. The nerve was fully identified and followed from its origin in the vagal nerve until its entry into the larynx. Along its path, the NRLN was completely enveloped by thyroid tissue from the right lobe in the region of the ZT immediately after its path became parallel to the thyroid artery (Figure 1). The patient did not present with laryngeal motor abnormalities or hypocalcaemia and is under treatment with oral thyroid replacement hormone.

## 3. Discussion

This case report describes the combination of the NRLN, a rare anatomical variation of the RLN present in less than 1% of people, with another variation not found in the consulted literature, which is the complete envelopment of the NRLN by thyroid tissue in the region of the ZT. Some authors have reported the occasional presence of a very close relationship between the nerve and the Berry ligament; for example, Kaisha et al. [5] found that it passed through the ligament in 7.4% of cases.

The ZT is present in most individuals who have been studied and has a constant relationship with the RLN close to its entry into the larynx. The RLN is typically located between the ZT and the trachea, posterior to the tubercle, which is an anatomical reference that can be used to help localize the nerve at this point [3]. The NRLN is typically associated with vascular malformation, although it has been found in isolation [6].

Several studies have reported that the safety of thyroid surgeries is directly related to the identification, dissection, and exposure of the RLN [7, 8]. The dissection technique requires broad understanding of the anatomy of the nerve, which includes knowledge of its anatomical and topographic variations [7]. Variations, such as those observed in this case, show that even with the most current knowledge of head and neck anatomy, surgeons can still encounter rare anatomical changes that can sometimes make the surgical procedure a challenge.

It is estimated that the rate of NRLN occurrence reaches 0.3% to 0.8% on the right side and 0.004% on the left side [7]. An NRLN enveloped by the ZT must be even rarer. The occurrence of an NRLN is often associated with increased risks of nerve injury. To minimize the risk of procedures, certain anatomical references are typically used during thyroid dissection procedures [2]. In previous reports, the ZT has been shown to play an important role in the prevention of iatrogenic injury. Due to its location and close relationship with the RLN, the ZT is an anatomical reference for locating the RLN during the important intraoperative period [8].

When an NRLN is present, careful and meticulous dissection around the tubercle should be performed for greater safety [6]. During the procedure, these recommendations were followed, making it possible to find the path of the NRLN, including its final course, in which it was enveloped by the right thyroid lobe in the region of the ZT.

Hamilton et al. [9] evaluated the impact of vagal nerve monitoring (VNM) during thyroidectomy and concluded that although the incidence of definitive paralysis of the RLN was comparable to the rates reported in the literature, the incidence of transient paralysis is lower with the use of VNM, which is able to safely predict paralysis of the RLN and to confirm its integrity at the end of the procedure. In our service, VNM is not routinely used, and it was not used in this case.

## 4. Conclusion

The inferior laryngeal nerve presents important anatomical variations. Nerve injuries can be avoided with knowledge of the anatomy and possible variations of the nerve. Identification and careful dissection using anatomical references such as the ITA, RLN, and tracheoesophageal sulcus can improve the success of procedures and reduce iatrogenic injuries.
